# Commercial price variation for common imaging studies

**DOI:** 10.1093/haschl/qxaf092

**Published:** 2025-04-30

**Authors:** Alexander P Philips, Christopher Whaley

**Affiliations:** Center for Advancing Health Policy Through Research (CAHPR), Brown School of Public Health, Providence, RI 02903, USA; Warren Alpert Medical School of Brown University, Providence, RI 02903, USA; Center for Advancing Health Policy Through Research (CAHPR), Brown School of Public Health, Providence, RI 02903, USA; Department of Health Services, Policy, and Practice, Brown School of Public Health, Providence, RI 02903, USA

**Keywords:** price variation, price transparency, commercial insurance, radiology

## Abstract

**Introduction:**

Commercial insurance payment rates for imaging studies have significant price variation, yet understanding this variation has been limited by lack of transparency and data limitations.

**Methods:**

Using newly available Transparency-in-Coverage insurer-posted data on negotiated rates, we analyzed price variation for the 2023 contract year across four major commercial insurers (Blue Cross Blue Shield, United, Cigna, and Aetna) for 30 imaging studies. Our analysis encompassed 12.7 million professional fee price points and 239 969 facility fee price points.

**Results:**

Our analysis revealed substantial variation in reimbursement rates. Key findings include greater variation in facility fees compared to professional fees, with facility coefficients of variation often 3 to 6 times higher than professional components. There was also substantial and inconsistent variation by payer. Geographic analysis revealed significant state-level variation, particularly in facility fees.

**Conclusion:**

These findings highlight the complex interplay of market dynamics and negotiating strategies in determining healthcare prices, with implications for policymakers, purchasers, and clinicians guiding patient care decisions.

## Introduction

Clinicians, patients, and purchasers face large variation in rates negotiated by commercial insurers, which provide health insurance coverage for approximately half the country. Unlike Medicare rates, commercial rates are established through a negotiation process that is not transparent, resulting in prices that are highly variable and opaque.^1,2^ Further, prices can vary depending on whether the contract is at the facility or physician level, with prices differing even within the same insurer's portfolio of plans.^3^ Lack of transparency limits the ability of regulators to monitor prices and employers, patients, and purchasers to impose market discipline on prices. The private market lacked meaningful price transparency for patients and purchasers until the recent implementation of Hospital Price Transparency and Transparency in Coverage (TiC) rules, which respectively require hospitals to post prices for 300 “shoppable” services and insurers to post their full set of negotiated rates. However, initial efforts to analyze the TiC data have been limited, largely due to data size and complexity. Price variation for common imaging studies has not been widely examined for large commercial payers since the implementation of TiC rules and is the objective of this study.

While existing studies show patients are unlikely to use price transparency data,^4-6^ these data can inform other decisions and policies. Clinicians play a key role in advising patients on care decisions and can benefit from understanding the wide range of prices offered by insurers to help them guide patients in navigating cost considerations and selecting affordable care options.^7^ Both purchasers and policymakers may also use price information to appropriately design initiatives that ensure health care affordability.^6,8^ Regulators can also use these data to monitor health care markets and design policies that limit the price consequences of provider concentration.^9,10^

To help fill this gap and inform these policies, in this study, we used TiC data to examine variation in commercial insurance payment rates for common imaging studies. Relative to other healthcare services, imaging studies are relatively homogenous.^11,12^ Thus, observed differences in prices are likely to reflect differences in negotiation leverage between both providers and payers. We also separately focused on price variation for professional fees, the amount paid to the radiologist to interpret the imaging study, and facility fees, the amount paid to the facility in which the imaging study occurs. Relative to other approaches which have identified substantial price variation in imaging studies,^13-16^ a key advantage of using the TiC data to examine these questions is the ability to specifically identify insurers. Further, TiC data allows us to comprehensively examine how insurer-specific factors translate into observed price differences for imaging studies*—*an analysis not possible with hospital transparency data, which are limited by incomplete reporting and focus only on hospital-based imaging prices, when many imaging services are performed in nonhospital settings.

## Data and methods

We examined TiC price data collected by a third-party vendor, ClarifyHealth, for common imaging studies from four large national insurers (Blue Cross Blue Shield [BCBS], United, Cigna, and Aetna), which collectively constitute 78% market share.^17^ Unlike the majority of prior work which utilizes hospital price data, we leverage TiC data's unique granularity and broader provider coverage to directly compare prices across all care settings (including hospitals) for major named insurers. Despite being released in July 2021, these data have seen limited use due to their structural complexity. However, recent research confirms high concordance between TiC data, hospital pricing data, and Marketscan commercial claims.^18,19^ Challenges with TiC data include “zombie rates” for inactive providers and redundant pricing files, resulting in over 1 trillion monthly price observations and 1 petabyte of data. To address these data challenge, Clarify links TiC data to Medicare fee-for-service and commercial insurance claims, covering about 270 million people.^20^ We analyzed commercial prices (excluding Medicare Advantage and Medicaid Managed Care) for providers with billed claims during the 2023 contract year for 30 common imaging studies. These data were downloaded in December 2024, but reflect the prior year's negotiated rates. Imaging studies were broadly categorized as either CT scan, mammogram, MRI, ultrasound, or X-ray studies. Prices reflect the “allowed amount,” which is the amount negotiated between an insurer and provider for a given Current Procedural Terminology (CPT) code (distinct from the “chargemaster” rate), and includes both payments from the insurer and patient cost-sharing responsibilities. We used weighted average negotiated prices across all plan types (PPO, HMO) as calculated by ClarifyHealth. This means if an insurer has enrollees in both PPO and HMO plans with different pricing structures, our analysis incorporates a weighted average of these prices.

For professional fee prices, our sample had approximately 12.7 million price points and 35 269 unique physicians. For facility fee prices, our sample included approximately 239 969 price points and 5057 facilities. We analyzed distributional differences in prices (mean, median, and percentiles), coefficients of variation, and price indices by payer. To construct price indices, we define procedure-specific volume weights for CPT codes (see Appendix 1), consistent with approaches used in previous literature.^21,22^

Several limitations should be noted in this analysis. First, we describe negotiated rates from four major commercial insurers, potentially missing important variation from smaller regional insurers and self-insured employers. Second, given its recency, these data are cross-sectional for the 2023 contract year and may not capture seasonal variations, longitudinal trends, or recent market changes. Third, we cannot track how much of the difference in payments translates to differences in physician compensation. Fourth, the price data does not account for quality differences, patient outcomes, or specific facility characteristics that might justify price variations. Fifth, we cannot stratify our price analysis by site-of-service principally due to differential reporting across payers.

Brown University Institutional Review Board deemed this cross-sectional study exempt from ethics review and informed consent because it was not human participant research. Data analysis was performed using R version 4.4.1.

## Results

Across all imaging studies examined, professional pricing has less price variation than facility pricing (Table 1). Facility coefficients of variation are often 3 to 6 times higher than their professional components. This difference is particularly pronounced in X-ray studies, where facility fees can vary by a factor of 6 or more. Advanced imaging modalities (CT and MRI) demonstrate more consistent pricing patterns with lower coefficients of variation in their facility fees (generally 1.2 to 2.0) compared to basic imaging like X-rays and ultrasounds.

**Table 1.** Average prices and price variation for 30 common imaging studies.

Procedure category	CPT code description	CPT code	Professional fees			Facility fees		
			Mean (SD)	Median (IQR)	Coef of variation	Mean (SD)	Median (IQR)	Coef of variation
CT scan	Head/brain scan without contrast	70 450	190 (164)	144 (100–225)	0.86	574 (1148)	292 (178–670)	2
	Chest scan without contrast	71 250	244 (205)	183 (129–288)	0.84	624 (1209)	334 (205–704)	1.94
	Chest scan with contrast	71 260	303 (261)	226 (162–345)	0.86	705 (1096)	468 (280–832)	1.56
	Chest blood vessel scan (angiography)	71 275	434 (373)	319 (237–480)	0.86	854 (1162)	569 (356–1084)	1.36
	Abdomen and pelvis scan without contrast	74 176	283 (224)	212 (162–324)	0.79	785 (1330)	496 (282–914)	1.69
	Abdomen and pelvis scan with contrast	74 177	445 (382)	336 (245–508)	0.86	1042 (1329)	730 (458–1245)	1.28
Mammogram	3D breast imaging (tomosynthesis), both breasts	77 063	69 (47)	55 (42–76)	0.68	237 (1573)	63 (40–109)	6.63
	Diagnostic mammogram with computer analysis, one breast	77 065	160 (115)	127 (96–179)	0.72	374 (1415)	195 (124–307)	3.79
	Diagnostic mammogram with computer analysis, both breasts	77 066	202 (146)	159 (121–227)	0.72	435 (1453)	236 (150–371)	3.34
	Screening mammogram with computer analysis, both breasts	77 067	167 (125)	131 (98–184)	0.75	361 (1387)	192 (123–305)	3.84
MRI scan	Brain MRI with and without contrast	70 553	619 (561)	447 (299–697)	0.91	1210 (1557)	792 (515–1487)	1.29
	Lower back (lumbar spine) MRI without contrast	72 148	380 (330)	278 (191–451)	0.87	920 (1283)	570 (348–1107)	1.4
	Lower extremity joint MRI without contrast	73 721	375 (306)	283 (196–442)	0.82	940 (1356)	582 (367–1126)	1.44
Ultrasound	Head and neck ultrasound	76 536	139 (113)	108 (78–153)	0.82	341 (1342)	167 (111–286)	3.94
	Complete breast ultrasound	76 641	128 (90)	101 (78–147)	0.7	391 (1580)	195 (120–304)	4.04
	Limited breast ultrasound	76 642	115 (83)	89 (68–132)	0.73	352 (1601)	158 (90–260)	4.55
	Complete abdominal ultrasound	76 700	156 (125)	120 (92–172)	0.8	348 (1311)	176 (116–301)	3.76
	Limited abdominal ultrasound	76 705	117 (92)	90 (70–129)	0.79	294 (1208)	141 (91–266)	4.11
	Transvaginal ultrasound (nonobstetric)	76 830	145 (121)	112 (83–160)	0.84	336 (1314)	174 (110–304)	3.91
	Complete pelvic ultrasound	76 856	138 (114)	106 (79–151)	0.83	312 (1124)	165 (105–292)	3.6
X-ray	Chest X-ray, single view	71 045	32 (28)	24 (17–35)	0.87	160 (1029)	48 (25–119)	6.41
	Chest X-ray, two views	71 046	43 (36)	32 (25–50)	0.83	197 (1253)	66 (36–142)	6.36
	Lower spine X-ray, 2–3 views	72 100	49 (44)	37 (28–54)	0.9	217 (1584)	66 (40–153)	7.31
	Shoulder X-ray	73 030	42 (35)	32 (24–47)	0.84	162 (1044)	56 (36–120)	6.44
	Hand X-ray	73 130	43 (35)	33 (24–49)	0.81	211 (1332)	61 (38–123)	6.31
	Knee X-ray, three views	73 562	48 (41)	37 (27–55)	0.85	204 (1192)	75 (38–140)	5.85
	Ankle X-ray	73 610	44 (40)	33 (25–49)	0.92	193 (1257)	61 (38–123)	6.53
	Foot X-ray	73 630	42 (36)	32 (24–46)	0.86	153 (949)	58 (36–119)	6.21
	Abdominal X-ray, single view	74 018	38 (31)	29 (22–42)	0.82	207 (1337)	61 (34–129)	6.45
	Bone density scan (DXA), spine/hips	77 080	70 (63)	53 (32–86)	0.9	299 (1455)	105 (54–226)	4.86

These data represent TiC data from ClarifyHealth, which aggregates price data for the 2023 contract year. Prices reflect the “allowed amount,” which is the amount negotiated between an insurer and provider for a given Current Procedural Terminology (CPT) code (distinct from the “chargemaster rate”). See Table 3 for this table disaggregated by commercial payer.

Source: Author's analysis.

Volume-weighted price indices by payer (Table 2) show BCBS consistently has higher payments across all imaging studies, with facility prices ranging from 31% to 85% above the market average. In contrast, Cigna and Aetna generally have lower-than-average prices across most imaging studies. United Healthcare presents a mixed pricing pattern, with professional fees close to the market average but notably lower facility fees, particularly for mammograms, at just 0.25 times the market average. Within service categories, the most extreme price variations appear in facility fees for X-rays, where BCBS charges 1.85 times the market average while Aetna charges only 0.32 times the market average—a 6-fold difference.

**Table 2.** Professional and facility price indices by payer for major imaging categories.

Payer	Professional Price Indices					
	CT scan	MRI scan	Mammogram	Ultrasound	X-ray	Overall
All Four Payers	1	1	1	1	1	1
Aetna	0.76	0.68	0.78	0.71	0.74	0.75
Blue Cross Blue Shield	1.12	1.13	1.08	1.11	1.1	1.11
Cigna	0.71	0.82	0.78	0.76	0.72	0.75
United Healthcare	0.96	0.92	1	1	0.99	0.97

Payer	Facility price indices					
	CT scan	MRI scan	Mammogram	Ultrasound	X-ray	Overall
All Four Payers	1	1	1	1	1	1
Aetna	0.83	0.81	0.54	0.46	0.32	0.67
Blue Cross Blue Shield	1.31	1.35	1.6	1.74	1.85	1.48
Cigna	0.71	0.68	0.68	0.7	0.36	0.65
United Healthcare	0.61	0.59	0.25	0.51	0.79	0.55

The methodology for calculating these volume-weighted price indices are shown in Appendix 1.

Source: Author's analysis.

Disaggregated analysis by both service and payer (Table 3) shows substantial price variation. The professional interquartile ratios were lowest for Cigna's CT Abdomen/Pelvis without contrast [CPT 74 176] (25th percentile: $137 to 75th percentile: $219) and highest for BCBS's DXA Bone Density Scan [CPT 77 080] (25th percentile: $34 to 75th percentile: $104). The facility interquartile ratios were lowest for the majority of UnitedHealthcare's imaging studies (with 17 of 30 studies showing an interquartile ratio of exactly 1) and highest for Aetna's CT Head without contrast [CPT 70 450] (25th percentile: $125 to 75th percentile: $700).

**Table 3.** Average prices and price variation for 30 common imaging studies by payer

Procedure category	Code description	Service code	Payer	Unique price points	Unique providers	Professional fees				Facility fees			
						Mean (SD)	Median (IQR)	Ratio of 75th to 25th %tiles	Coef of variation	Mean (SD)	Median (IQR)	Ratio of 75th to 25th %tiles	Coef of variation
CT scan	Head/Brain scan without contrast	70 450	All Four Payers	520 237	26 387	190 (164)	144 (100–225)	2.25	0.86	574 (1148)	292 (178–670)	3.76	2
			Aetna	62 618	13 794	136 (108)	103 (71–164)	2.31	0.79	506 (552)	257 (125–700)	5.61	1.09
			BlueCross BlueShield	297 519	24 212	217 (177)	162 (108–266)	2.46	0.82	773 (1771)	462 (238–777)	3.27	2.29
			Cigna	56 008	18 599	141 (74)	129 (89–175)	1.96	0.53	364 (318)	238 (170–501)	2.95	0.87
			UnitedHealthcare	104 092	19 657	174 (169)	141 (105–200)	1.92	0.97	273 (59)	281 (281–281)	1	0.22
	Chest scan without contrast	71 250	All Four Payers	513 670	26 757	244 (205)	183 (129–288)	2.24	0.84	624 (1209)	334 (205–704)	3.44	1.94
			Aetna	63 132	14 021	166 (126)	126 (91–186)	2.04	0.76	537 (551)	303 (156–742)	4.77	1.03
			BlueCross BlueShield	295 558	24 560	278 (236)	206 (141–316)	2.23	0.85	828 (1823)	491 (262–795)	3.04	2.2
			Cigna	56 070	18 763	184 (94)	169 (117–227)	1.94	0.51	435 (307)	304 (255–628)	2.46	0.7
			UnitedHealthcare	98 910	19 309	227 (165)	187 (126–270)	2.15	0.73	297 (67)	313 (313–313)	1	0.23
	Chest scan with contrast	71 260	All Four Payers	517 772	27 096	303 (261)	226 (162–345)	2.13	0.86	705 (1096)	468 (280–832)	2.97	1.56
			Aetna	62 965	14 079	210 (175)	159 (114–233)	2.04	0.83	569 (532)	372 (201–762)	3.79	0.94
			BlueCross BlueShield	299 007	24 945	343 (304)	254 (181–381)	2.11	0.89	980 (1675)	660 (409–1047)	2.56	1.71
			Cigna	56 501	18 996	222 (115)	205 (144–273)	1.9	0.52	497 (315)	375 (285–707)	2.48	0.63
			UnitedHealthcare	99 299	19 626	286 (183)	235 (170–344)	2.03	0.64	450 (111)	468 (468–468)	1	0.25
	Chest blood vessel scan (angiography)	71 275	All Four Payers	493 663	25 502	434 (373)	319 (237–480)	2.03	0.86	854 (1162)	569 (356–1084)	3.05	1.36
			Aetna	59 886	13 330	283 (209)	214 (160–310)	1.94	0.74	711 (632)	494 (275–990)	3.61	0.89
			BlueCross BlueShield	285 981	23 486	488 (433)	349 (260–514)	1.98	0.89	1086 (1643)	725 (461–1236)	2.68	1.51
			Cigna	53 735	17 829	335 (171)	320 (217–404)	1.87	0.51	714 (336)	633 (542–857)	1.58	0.47
			UnitedHealthcare	94 061	18 412	425 (292)	350 (247–509)	2.06	0.69	396 (181)	375 (272–500)	1.83	0.46
	Abdomen and pelvis scan without contrast	74 176	All Four Payers	501 607	26 344	283 (224)	212 (162–324)	2	0.79	785 (1330)	496 (282–914)	3.24	1.69
			Aetna	60 638	13 657	240 (177)	170 (147–266)	1.81	0.74	629 (617)	399 (226–833)	3.68	0.98
			BlueCross BlueShield	293 786	24 279	316 (257)	239 (175–347)	1.98	0.81	1038 (1964)	620 (388–1066)	2.75	1.89
			Cigna	52 078	17 796	194 (98)	170 (137–219)	1.59	0.51	531 (314)	409 (350–707)	2.02	0.59
			UnitedHealthcare	95 105	18 668	260 (158)	217 (159–314)	1.98	0.61	502 (262)	462 (351–599)	1.71	0.52
	Abdomen and pelvis scan with contrast	74 177	All Four Payers	537 560	28 433	445 (382)	336 (245–508)	2.07	0.86	1042 (1329)	730 (458–1245)	2.72	1.28
			Aetna	64 600	14 601	367 (253)	267 (227–415)	1.83	0.69	841 (704)	631 (364–1129)	3.11	0.84
			BlueCross BlueShield	314 438	26 241	489 (425)	357 (264–538)	2.03	0.87	1335 (1860)	913 (555–1557)	2.8	1.39
			Cigna	55 394	19 227	297 (161)	265 (201–346)	1.72	0.54	767 (346)	707 (591–1144)	1.94	0.45
			UnitedHealthcare	103 128	20 498	439 (368)	363 (238–535)	2.25	0.84	750 (418)	654 (443–978)	2.21	0.56
Mammogram	3D breast imaging (tomosynthesis), both breasts	77 063	All Four Payers	268 059	16 178	69 (47)	55 (42–76)	1.8	0.68	237 (1573)	63 (40–109)	2.72	6.63
			Aetna	31 570	7614	54 (38)	39 (34–56)	1.67	0.71	83 (65)	61 (40–105)	2.66	0.79
			BlueCross BlueShield	155 123	14 798	75 (53)	59 (47–82)	1.74	0.71	449 (2393)	69 (45–124)	2.74	5.33
			Cigna	27 509	10 707	53 (24)	46 (38–62)	1.62	0.46	110 (60)	100 (57–117)	2.06	0.54
			UnitedHealthcare	53 857	11 644	68 (35)	59 (43–79)	1.83	0.51	31 (22)	26 (24–26)	1.1	0.7
	Diagnostic mammogram with computer analysis, one breast	77 065	All Four Payers	193 578	12 334	160 (115)	127 (96–179)	1.87	0.72	374 (1415)	195 (124–307)	2.48	3.79
			Aetna	23 818	5897	127 (81)	96 (85–135)	1.6	0.64	221 (155)	175 (107–280)	2.62	0.7
			BlueCross BlueShield	110 838	11 306	171 (134)	131 (100–183)	1.83	0.79	554 (2052)	215 (156–337)	2.16	3.71
			Cigna	19 486	7881	128 (62)	112 (89–146)	1.65	0.48	287 (152)	241 (176–327)	1.86	0.53
			UnitedHealthcare	39 436	8860	163 (83)	141 (103–192)	1.85	0.51	108 (33)	96 (87–113)	1.29	0.31
	Diagnostic mammogram with computer analysis, both breasts	77 066	All Four Payers	192 184	12 254	202 (146)	159 (121–227)	1.88	0.72	435 (1453)	236 (150–371)	2.47	3.34
			Aetna	23 461	5815	161 (103)	123 (107–174)	1.62	0.64	277 (196)	208 (136–344)	2.53	0.71
			BlueCross BlueShield	110 359	11 223	216 (170)	165 (124–232)	1.87	0.79	626 (2113)	263 (184–432)	2.35	3.38
			Cigna	19 379	7863	161 (78)	141 (113–184)	1.63	0.48	374 (191)	328 (251–425)	1.69	0.51
			UnitedHealthcare	38 985	8804	207 (107)	177 (131–242)	1.84	0.52	132 (38)	123 (112–129)	1.15	0.28
	Screening mammogram with computer analysis, both breasts	77 067	All Four Payers	281 714	16 805	167 (125)	131 (98–184)	1.88	0.75	361 (1387)	192 (123–305)	2.48	3.84
			Aetna	33 977	8196	130 (85)	98 (86–138)	1.6	0.66	222 (163)	161 (108–278)	2.57	0.73
			BlueCross BlueShield	163 670	15 345	181 (146)	136 (101–188)	1.85	0.81	534 (2035)	221 (153–341)	2.23	3.81
			Cigna	28 035	10 908	130 (62)	114 (91–149)	1.64	0.48	274 (142)	247 (148–303)	2.04	0.52
			UnitedHealthcare	56 032	12 070	167 (87)	144 (105–196)	1.86	0.52	111 (32)	101 (92–115)	1.25	0.29
MRI scan	Brain MRI with and without contrast	70 553	All Four Payers	380 432	19 448	619 (561)	447 (299–697)	2.33	0.91	1210 (1557)	792 (515–1487)	2.89	1.29
			Aetna	44 339	9962	390 (370)	268 (213–427)	2	0.95	930 (831)	652 (353–1321)	3.74	0.89
			BlueCross BlueShield	218 204	17 814	695 (645)	498 (333–750)	2.25	0.93	1693 (2238)	1107 (716–1874)	2.62	1.32
			Cigna	41 433	13 694	519 (325)	433 (275–700)	2.54	0.63	843 (402)	720 (616–957)	1.55	0.48
			UnitedHealthcare	76 456	14 420	590 (434)	469 (329–692)	2.1	0.74	803 (249)	792 (792–792)	1	0.31
	Lower back (lumbar spine) MRI without contrast	72 148	All Four Payers	383 475	19 554	380 (330)	278 (191–451)	2.36	0.87	920 (1283)	570 (348–1107)	3.18	1.4
			Aetna	45 114	10 066	273 (233)	220 (162–301)	1.86	0.85	792 (765)	479 (237–1128)	4.77	0.97
			BlueCross BlueShield	222 328	17 951	429 (381)	315 (201–523)	2.6	0.89	1187 (1832)	797 (474–1223)	2.58	1.54
			Cigna	41 054	13 697	304 (176)	265 (165–401)	2.43	0.58	612 (394)	446 (368–888)	2.41	0.64
			UnitedHealthcare	74 979	14 260	340 (241)	275 (192–400)	2.08	0.71	531 (148)	531 (531–531)	1	0.28
	Lower extremity joint MRI without contrast	73 721	All Four Payers	268 579	13650	375 (306)	283 (196–442)	2.26	0.82	940 (1356)	582 (367–1126)	3.07	1.44
			Aetna	32 573	7248	265 (194)	219 (167–299)	1.79	0.73	796 (764)	494 (246–1127)	4.58	0.96
			BlueCross BlueShield	156 605	12 599	423 (354)	321 (211–516)	2.45	0.84	1256 (1949)	833 (503–1272)	2.53	1.55
			Cigna	27 795	9393	299 (167)	267 (175–384)	2.2	0.56	635 (409)	457 (391–925)	2.37	0.64
			UnitedHealthcare	51 606	9941	340 (219)	270 (198–411)	2.07	0.64	430 (163)	393 (393–393)	1	0.38
Ultrasound	Head and neck ultrasound	76 536	All Four Payers	442 672	23 783	139 (113)	108 (78–153)	1.96	0.82	341 (1342)	167 (111–286)	2.57	3.94
			Aetna	54 690	12 232	96 (79)	68 (58–101)	1.73	0.82	157 (116)	129 (81–200)	2.47	0.74
			BlueCross BlueShield	252 150	21 750	153 (133)	114 (88–158)	1.81	0.87	633 (2121)	279 (193–437)	2.27	3.35
			Cigna	47 783	16 584	107 (55)	92 (72–127)	1.75	0.52	258 (119)	227 (207–297)	1.43	0.46
			UnitedHealthcare	88 049	17 347	143 (79)	124 (87–171)	1.97	0.55	147 (84)	117 (117–117)	1	0.57
	Complete breast ultrasound	76 641	All Four Payers	145 902	9035	128 (90)	101 (78–147)	1.89	0.7	391 (1580)	195 (120–304)	2.53	4.04
			Aetna	19 969	4602	105 (91)	80 (63–108)	1.71	0.86	193 (157)	147 (94–238)	2.52	0.81
			BlueCross BlueShield	83 810	8180	139 (97)	108 (88–156)	1.78	0.7	649 (2381)	242 (175–366)	2.1	3.67
			Cigna	14 991	5964	100 (45)	88 (72–117)	1.61	0.45	279 (138)	233 (192–474)	2.46	0.5
			UnitedHealthcare	27 132	6232	124 (74)	101 (73–148)	2.04	0.59	236 (98)	222 (159–297)	1.87	0.42
	Limited breast ultrasound	76 642	All Four Payers	254 920	14 836	115 (83)	89 (68–132)	1.95	0.73	352 (1601)	158 (90–260)	2.88	4.55
			Aetna	30 587	7243	90 (71)	66 (55–92)	1.67	0.79	152 (112)	115 (73–194)	2.67	0.74
			BlueCross BlueShield	149 549	13 642	126 (93)	97 (73–136)	1.86	0.74	586 (2346)	204 (140–337)	2.41	4
			Cigna	26 214	9896	86 (41)	74 (60–100)	1.65	0.48	199 (103)	182 (132–227)	1.72	0.52
			UnitedHealthcare	48 570	10 553	112 (67)	95 (65–132)	2.03	0.6	204 (89)	192 (144–246)	1.71	0.44
	Complete abdominal ultrasound	76 700	All Four Payers	455 315	23 522	156 (125)	120 (92–172)	1.88	0.8	348 (1311)	176 (116–301)	2.6	3.76
			Aetna	56 326	12 390	104 (82)	76 (62–114)	1.84	0.79	168 (140)	124 (77–218)	2.81	0.83
			BlueCross BlueShield	262 205	21 583	175 (143)	128 (100–184)	1.84	0.82	613 (2031)	281 (196–448)	2.29	3.31
			Cigna	50 275	16 691	119 (57)	105 (81–137)	1.7	0.48	279 (136)	239 (217–313)	1.44	0.49
			UnitedHealthcare	86 509	16 880	152 (104)	125 (87–186)	2.13	0.69	173 (70)	154 (154–154)	1	0.41
	Limited abdominal ultrasound	76 705	All Four Payers	524 090	27 387	117 (92)	90 (70–129)	1.86	0.79	294 (1208)	141 (91–266)	2.93	4.11
			Aetna	64 030	14 162	80 (62)	58 (48–89)	1.85	0.77	128 (115)	95 (59–165)	2.79	0.9
			BlueCross BlueShield	301 181	25 155	131 (107)	94 (75–139)	1.86	0.82	529 (1858)	260 (161–402)	2.5	3.51
			Cigna	57 369	19 253	88 (44)	78 (59–102)	1.72	0.5	200 (96)	169 (163–227)	1.39	0.48
			UnitedHealthcare	101 510	20 026	117 (66)	98 (71–139)	1.97	0.57	135 (77)	109 (109–109)	1	0.57
	Transvaginal ultrasound (nonobstetric)	76 830	All Four Payers	469 934	24 071	145 (121)	112 (83–160)	1.92	0.84	336 (1314)	174 (110–304)	2.76	3.91
			Aetna	58 327	12 727	101 (75)	73 (62–106)	1.69	0.74	165 (127)	126 (75–215)	2.87	0.77
			BlueCross BlueShield	267 020	22 095	159 (145)	116 (91–165)	1.81	0.91	583 (2024)	280 (176–443)	2.51	3.47
			Cigna	51 018	16 929	112 (54)	98 (78–131)	1.69	0.49	250 (119)	215 (196–284)	1.45	0.48
			UnitedHealthcare	93 569	17 626	150 (82)	126 (91–181)	1.99	0.54	161 (78)	135 (135–135)	1	0.49
	Complete pelvic ultrasound	76 856	All Four Payers	473 778	24 101	138 (114)	106 (79–151)	1.92	0.83	312 (1124)	165 (105–292)	2.78	3.6
			Aetna	58 852	12 796	94 (74)	68 (56–98)	1.73	0.79	152 (131)	110 (70–196)	2.79	0.86
			BlueCross BlueShield	269 688	22 163	154 (136)	112 (88–157)	1.79	0.88	527 (1713)	269 (180–427)	2.37	3.25
			Cigna	51 193	16 937	106 (50)	94 (73–122)	1.68	0.47	227 (108)	194 (177–258)	1.46	0.48
			UnitedHealthcare	94 045	17 700	138 (74)	119 (84–164)	1.96	0.54	182 (73)	163 (163–163)	1	0.4
X-ray	Chest X-ray, single view	71 045	All Four Payers	540 881	28 920	32 (28)	24 (17–35)	2.02	0.87	160 (1029)	48 (25–119)	4.7	6.41
			Aetna	60 164	13 785	24 (21)	17 (14–25)	1.79	0.91	49 (94)	31 (19–53)	2.73	1.9
			BlueCross BlueShield	318 140	26 716	34 (31)	25 (20–39)	1.95	0.89	285 (1518)	96 (40–180)	4.52	5.33
			Cigna	56 180	19 435	22 (12)	19 (15–28)	1.88	0.54	55 (31)	47 (28–58)	2.09	0.57
			UnitedHealthcare	106 397	21 039	33 (25)	26 (18–40)	2.29	0.77	180 (116)	171 (100–241)	2.41	0.65
	Chest X-ray, two views	71 046	All Four Payers	549 078	29 245	43 (36)	32 (25–50)	2.02	0.83	197 (1253)	66 (36–142)	3.97	6.36
			Aetna	62 461	14 121	31 (24)	23 (20–32)	1.6	0.77	64 (92)	43 (26–75)	2.93	1.43
			BlueCross BlueShield	321 837	26 980	48 (41)	35 (27–52)	1.93	0.87	348 (1849)	108 (59–192)	3.25	5.31
			Cigna	56 290	19 556	31 (16)	27 (21–38)	1.78	0.5	68 (38)	62 (36–75)	2.09	0.56
			UnitedHealthcare	108 490	21 276	43 (27)	35 (23–54)	2.31	0.62	178 (107)	169 (100–238)	2.38	0.6
	Lower spine X-ray, 2–3 views	72 100	All Four Payers	499 658	25 857	49 (44)	37 (28–54)	1.9	0.9	217 (1584)	66 (40–153)	3.78	7.31
			Aetna	60 429	13 422	37 (56)	26 (23–37)	1.61	1.52	62 (89)	43 (27–75)	2.74	1.43
			BlueCross BlueShield	286 055	23 725	54 (48)	40 (31–57)	1.86	0.89	449 (2514)	159 (81–230)	2.83	5.6
			Cigna	54 189	18 094	37 (18)	33 (25–44)	1.72	0.49	86 (44)	75 (48–103)	2.13	0.51
			UnitedHealthcare	98 985	18 840	48 (28)	40 (29–58)	1.98	0.58	88 (96)	48 (48–48)	1	1.09
	Shoulder X-ray	73 030	All Four Payers	515 580	26 437	42 (35)	32 (24–47)	1.95	0.84	162 (1044)	56 (36–120)	3.37	6.44
			Aetna	62 155	13 688	31 (30)	22 (19–31)	1.64	0.99	56 (93)	37 (23–64)	2.71	1.67
			BlueCross BlueShield	295 633	24 295	46 (40)	36 (26–49)	1.87	0.87	315 (1636)	110 (68–192)	2.8	5.19
			Cigna	55 555	18 423	31 (16)	27 (21–37)	1.75	0.51	71 (38)	64 (37–77)	2.09	0.54
			UnitedHealthcare	102 237	19 370	42 (25)	34 (26–49)	1.88	0.59	75 (76)	44 (44–44)	1	1.01
	Hand X-ray	73 130	All Four Payers	498 973	25 566	43 (35)	33 (24–49)	2.01	0.81	211 (1332)	61 (38–123)	3.27	6.31
			Aetna	59 977	13 258	32 (28)	23 (20–33)	1.64	0.89	58 (87)	40 (25–68)	2.73	1.5
			BlueCross BlueShield	286 032	23 504	48 (40)	36 (27–51)	1.88	0.84	435 (2082)	118 (73–200)	2.73	4.78
			Cigna	54 107	18 018	32 (17)	28 (22–39)	1.76	0.52	81 (42)	70 (46–93)	2.01	0.52
			UnitedHealthcare	98 857	18 709	43 (26)	36 (25–51)	2.07	0.62	75 (78)	42 (42–42)	1	1.05
	Knee X-ray, three views	73 562	All Four Payers	488 612	24 696	48 (41)	37 (27–55)	2.01	0.85	204 (1192)	75 (38–140)	3.7	5.85
			Aetna	58 899	12 902	36 (42)	25 (22–36)	1.6	1.19	62 (73)	44 (27–75)	2.73	1.19
			BlueCross BlueShield	280 482	22 719	54 (46)	41 (30–58)	1.9	0.86	374 (1778)	124 (79–208)	2.63	4.75
			Cigna	52 808	17 371	36 (19)	31 (24–43)	1.77	0.53	86 (48)	75 (45–99)	2.22	0.55
			UnitedHealthcare	96 423	18 094	47 (28)	39 (29–55)	1.92	0.58	178 (105)	162 (110–225)	2.05	0.59
	Ankle X-ray	73 610	All Four Payers	498 854	25 536	44 (40)	33 (25–49)	1.99	0.92	193 (1257)	61 (38–123)	3.24	6.53
			Aetna	59 997	13 258	33 (49)	23 (20–33)	1.63	1.48	58 (86)	40 (25–70)	2.77	1.5
			BlueCross BlueShield	286 748	23 496	48 (44)	35 (28–53)	1.91	0.92	388 (1963)	119 (74–200)	2.72	5.06
			Cigna	53 678	17 842	32 (17)	28 (22–39)	1.77	0.52	76 (42)	68 (39–83)	2.11	0.54
			UnitedHealthcare	98 431	18 676	43 (25)	36 (26–52)	1.96	0.58	74 (79)	41 (41–41)	1	1.08
	Foot X-ray	73 630	All Four Payers	507 314	26 045	42 (36)	32 (24–46)	1.96	0.86	153 (949)	58 (36–119)	3.34	6.21
			Aetna	60 859	13 479	31 (32)	22 (19–31)	1.62	1.05	55 (87)	37 (24–66)	2.73	1.56
			BlueCross BlueShield	291 682	23 961	46 (40)	33 (26–50)	1.93	0.88	294 (1488)	112 (70–191)	2.75	5.06
			Cigna	54 630	18 200	31 (16)	27 (21–37)	1.7	0.5	72 (37)	63 (38–76)	2.03	0.52
			UnitedHealthcare	100 143	19 089	42 (29)	34 (25–49)	2.01	0.71	72 (77)	42 (42–42)	1	1.06
	Abdominal X-ray, single view	74 018	All Four Payers	512 546	27 347	38 (31)	29 (22–42)	1.97	0.82	207 (1337)	61 (34–129)	3.81	6.45
			Aetna	57 159	13 131	28 (23)	21 (18–30)	1.66	0.81	58 (86)	40 (24–67)	2.74	1.48
			BlueCross BlueShield	300 334	25 251	42 (36)	31 (24–44)	1.87	0.85	371 (1950)	103 (55–181)	3.28	5.25
			Cigna	53 353	18 423	28 (14)	24 (19–34)	1.77	0.5	65 (34)	55 (35–78)	2.2	0.52
			UnitedHealthcare	101 700	19 952	38 (24)	30 (20–46)	2.26	0.65	181 (109)	172 (107–241)	2.26	0.6
	Bone density scan (DXA), spine/hips	77 080	All Four Payers	278 039	16 062	70 (63)	53 (32–86)	2.68	0.9	299 (1455)	105 (54–226)	4.17	4.86
			Aetna	35 258	8160	53 (56)	35 (26–63)	2.43	1.07	102 (113)	64 (38–117)	3.1	1.11
			BlueCross BlueShield	160 815	14 674	80 (71)	61 (34–104)	3.01	0.89	544 (2173)	199 (108–316)	2.93	3.99
			Cigna	27 952	10 503	55 (29)	48 (33–72)	2.2	0.52	91 (43)	79 (70–103)	1.46	0.47
			UnitedHealthcare	54 014	11 341	60 (44)	47 (32–71)	2.2	0.74	246 (116)	245 (180–303)	1.69	0.47

These data represent TiC data from ClarifyHealth, which aggregates price data for the 2023 contract year. Prices reflect the “allowed amount,” which is the amount negotiated between an insurer and provider for a given Current Procedural Terminology (CPT) code (distinct from the “chargemaster rate”).

Source: Author's analysis.

**Figure 1.**
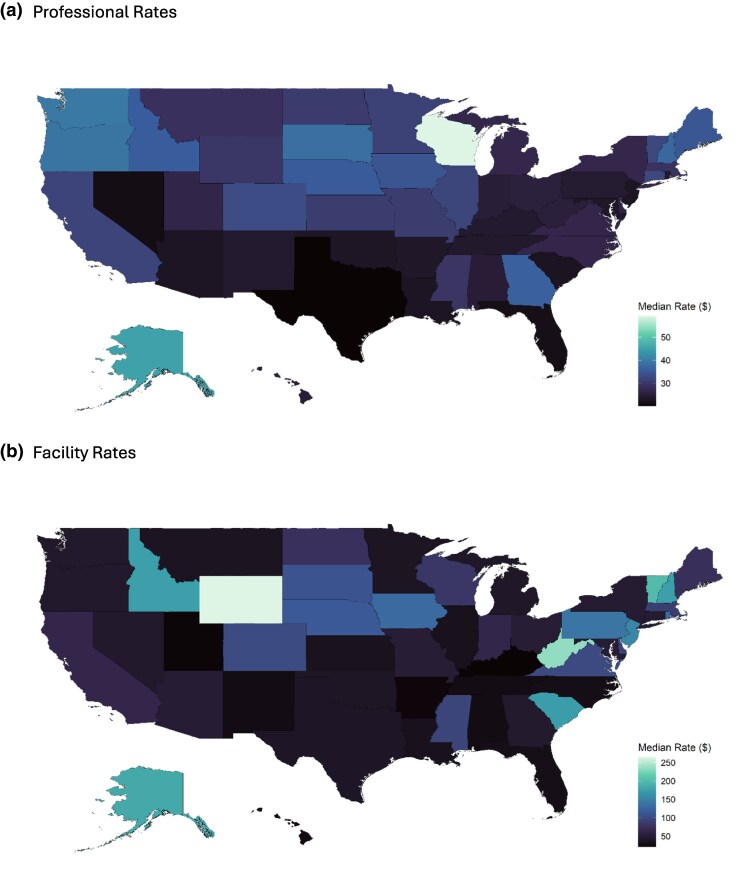
Map of median rates by state for chest X-ray [CPTs 71 045 and 71 046]. Source: author's analysis. (A) Professional rates. (B) Facility rates.

As a descriptive example of price variation for imaging studies by geography, Figure 1 maps the variation in prices for chest X-rays across states. Professional prices for chest X-rays are relatively low and show limited variation across states, but facility prices are higher and have an upper limit of approximately 5 times its lower bound across states. Similar findings were present for other procedures.

## Discussion

The findings of this study highlight significant variation in commercial pricing for common imaging studies, with facility prices exhibiting greater variability, especially for X-ray studies. We observe not just geographic variation in commercial prices, but also payer-specific variation across named insurers that suggest unique bargaining strategies, and extend prior work using hospital-posted data by including both hospital and nonhospital providers. The higher prices observed for BCBS likely reflect their market power and strategic decision to maintain broad provider networks, which typically require higher reimbursement rates to attract and retain providers.^23,24^ Cigna's consistently lower prices across imaging studies suggest a successful implementation of selective contracting with providers who accept lower reimbursement rates in exchange for higher patient volume.^23^ Aetna's consistently lower prices, particularly in facility fees, suggests an aggressive negotiating stance with providers and possibly a willingness to exclude higher-cost facilities from their networks,^25^ even if this means more limited geographic coverage. United Healthcare's unique pattern of average professional fees but markedly lower facility fees, particularly in mammography, may reflect a vertical integration strategy through its subsidiary, Optum, where it can better control facility costs through direct ownership while maintaining competitive physician compensation to ensure network adequacy.^26^

The pricing patterns observed in part reflect different negotiation strategies and market positioning. While our analysis examines variation across major insurers, significant heterogeneity exists within individual insurers’ networks. For example, different plans (including employer-specific plans) from the same insurer can have substantially different prices even within the same hospital or for the same physician.^27,28^ This within-insurer variation likely contributes to the overall price dispersion observed in our study.

Our findings of greater variation in facility fees for X-rays compared to advanced imaging like MRI and CT may reflect, in part, differences in site-of-service utilization. X-rays can be performed across multiple settings (hospitals, ambulatory surgical centers, and physician offices), while advanced imaging is more commonly performed in hospital settings. Recent studies using Transparency-in-Coverage data have documented substantial site-of-service price differentials for other services. Wang et al.^29^ found facility fees for colonoscopies were approximately 55% higher in hospitals compared to ambulatory surgical centers. Similar patterns exist for imaging services, with BCBS data showing hospital outpatient department prices for imaging 2–3 times higher than physician office prices, with these differentials increasing over time.^30^ While our current analysis focuses on payer-specific variation rather than site-of-service differences, the observed price patterns—particularly the wider variation in X-ray facility fees—may reflect these site-of-service dynamics. Future research should examine how imaging prices vary across different care settings and how site-neutral payment policies might affect commercial market prices.

The cost burden on patients and insurers can differ widely depending on the location and the service provided, potentially exacerbating healthcare inequities. The state-level variation in prices further underscores the potential influence of local market dynamics, such as provider market concentration, insurer market power, cost of living adjustments, state-specific regulatory environments, and local practice patterns—with facility fees showing particularly pronounced geographic disparities compared to the more uniform professional component pricing. Outlier states (such as Wisconsin and Alaska) with higher than average professional prices may be explained by strong physician practices with increased negotiating leverage^1^ or Medicare payment policy allowing for geographic payment adjustment.^31^

This study reveals how novel TiC data may inform policies that improve the efficiency of the US health care system. Future work should extend this study and examine the underlying causes of price variation in imaging studies. Existing studies show minimal relationship between prices and care quality or efficiency.^32^ The disconnect between pricing and quality metrics is particularly noteworthy in imaging studies, where standardization of equipment and protocols should theoretically lead to more uniform pricing structures.^11,33^ Even among these relatively standardized procedures, we observe inconsistent patterns of commercial insurance price variation. If price variation reflects clinical or perceived quality variation, purchasers and policymakers must find the balance between receiving higher-quality care and spending financial resources elsewhere. However, if price variation is driven by consolidation or anticompetitive contracting, then regulators should design policies that ensure competitive healthcare markets. The factors determining price variation are likely somewhere in the middle of these two possibilities, but likely skewed towards the latter in the case of imaging studies where quality should be relatively constant.

## Supplementary Material

qxaf092_Supplementary_Data
