# Supplementary material for: Commercial price variation for common imaging studies
Source: Health Aff Sch. 2025 Apr 30;3(5):qxaf092. doi: 10.1093/haschl/qxaf092 (PMC12097484; doi:10.1093/haschl/qxaf092)
Supplement: qxaf092_Supplementary_Data [file qxaf092_supplementary_data.zip › Appendix 1.docx]

Appendix 1: Price Index Construction

Following previous methods of constructing price indices (e.g. Dunn, Shapiro, and Liebman 2013; Neprash et al. 2015), we define procedure-specific weights for CPT code c as the following and where x is the number of CPT codes in the Procedure Category we are examining (for example, x = 6 for CT Scans):

$$w_{c}=\frac{price_{c}* q_{c}}{\sum_{c = 1}^{C = x} \left( price_{c}* q_{c} \right)}$$

And for each insurer b, we define price index as:

$$index_{b}= \sum_{b = 1}^{B = 4} \frac{price_{cb}}{price_{c}}* w_{c}$$
